# Optimizing psychotherapy dosage for comorbid depression and personality disorders (PsyDos): a pragmatic randomized factorial trial using schema therapy and short-term psychodynamic psychotherapy

**DOI:** 10.1186/s12888-018-1829-1

**Published:** 2018-08-07

**Authors:** Marit Kool, Henricus L. Van, Anna Bartak, Saskia C. M. de Maat, Arnoud Arntz, Johanna W. van den Eshof, Jaap Peen, Matthijs Blankers, Judith E. Bosmans, Jack J. M. Dekker

**Affiliations:** 1 Arkin Mental Health Care, Domselaerstraat 128, 1093 MB Amsterdam, the Netherlands; 2 Department of Clinical Psychology, University of Amsterdam, Amsterdam, the Netherlands; 3 Department of Research, Arkin Mental Health Care, Amsterdam, the Netherlands; 4 Amsterdam UMC, Location AMC, Department of Psychiatry, University of Amsterdam, Amsterdam, the Netherlands; 5 Trimbos Institute – Netherlands Institute of Mental Health and Addiction, Utrecht, the Netherlands; 6 Department of Health Sciences, Faculty of Earth & Life Sciences, Free University Amsterdam, Amsterdam Public Health Research Institute, Amsterdam, The Netherlands; 7 Department of Clinical Psychology, VU University of Amsterdam, Amsterdam, the Netherlands

**Keywords:** Dosage, Depression, Personality, Schema, Psychodynamic, Randomized controlled trial, Economic evaluation

## Abstract

**Background:**

Patients with comorbid depression and personality disorders suffer from a heavy disease burden while tailored treatment options are limited, accounting for a high psychological and economic burden. Little is known about the effect of treatment dosage and type of psychotherapy for this specific co-morbid patient population, in terms of treatment-effect and cost-effectiveness. This study aims to compare treatment outcome of 25 versus 50 individual therapy sessions in a year. We expect the 50-session condition to be more effective in treating depression and maintaining the effect. Secondary objectives will be addressed in order to find therapy-specific and non-specific mechanisms of change.

**Methods:**

In a mono-center pragmatic randomized controlled trial with a 2 × 2 factorial design, 200 patients with a depressive disorder and personality disorder(s) will be included. Patients will be recruited from a Dutch mental health care institute for personality disorders. They will be randomized over therapy dosage (25 vs 50 sessions in a year) and type of therapy (schema therapy vs short-term psychodynamic supportive psychotherapy). The primary clinical outcome measure will be depression severity and remission. Changes in personality functioning and quality of life will be investigated as secondary outcomes. A priori postulated effect moderators and mediators will be collected as well. All patients are assessed at baseline and at 1, 2, 3, 6, 9–12 months (end of therapy) and at follow up (6 and 12 months after end of treatment). Alongside the trial, an economic evaluation will be conducted. Costs will be collected from a societal perspective.

**Discussion:**

This trial will be the first to compare two psychotherapy dosages in patients with both depression and personality disorders. Insight in the effect of treatment dosage for this patient group will contribute to both higher treatment effectiveness and lower costs. In addition, this study will contribute to the limited evidence base on treating patients with both depression and personality disorders. Understanding the processes that account for the therapeutic changes could help to gain insight in what works for whom.

**Trial registration:**

This trial has been registered on July 20th 2016, Netherlands Trial Register, part of the Dutch Cochrane Centre (NTR5941).

## Background

Depressive disorders and personality disorders (PD) often co-occur [1–3]. Both depressive disorders and personality disorders are highly invalidating conditions and they represent a tremendous financial burden on society [4–7]. A meta-analysis on the influence of PD on outcome of depression showed that treatment is two times less effective for depressed patients with PD than those without PD [8].This review is based on pretreatment predictor analyses of outcomes in 34 studies with broad samples of depressed patients. Vice versa, research on the impact of a comorbid depressive disorder on recovery of PD is scarce. In an randomized clinical trial (RCT) with mainly cluster-C patients, co-occurring depressions were associated with lower recovery rates at 3 year follow-up (*p* = 0.01). This effect disappeared when controlled for general severity [3], suggesting that not the depressive disorder but high general severity at baseline is a negative predictor for success. In a study on the impact of dysthymic disorder on the outcome in PD patients however, a dysthymic disorder at baseline was related to the persistence of PD diagnoses at 2 years, especially for Borderline PD and Avoidant PD [9].

Despite the urgent need to extend our knowledge of treatment effect in the co-morbid group of patients with both depression and PD, until recently available empirically supported treatments (EST’s) have addressed either depression symptoms or personality pathology. In recent years more integrated therapies such as Schema Therapy (ST) and Short-term Psychodynamic Supportive Psychotherapy (SPSP) have been developed, focusing on depression in relation to inter- and intrapersonal patterns while taking into account etiologic long standing personality vulnerabilities.

Of these, psychodynamic therapies are promising as they are directed at long standing inter- and intrapersonal patterns relating to depression [10]. Evidence for the psychodynamic approach in depression has been demonstrated in a number of meta-analyses [11, 12] and in recent empirical studies [13, 14].

In addition, ST is an integrated psychotherapy originally designed to treat PD and chronic axis-I disorders using techniques from cognitive behavioral therapy, psychoanalytic object relations theory, attachment theory and Gestalt therapy [15]. A study on ST in patients with PD shows a concurrent improvement in depressive symptoms [16] indicating ST might be effective in PD patients with comorbid depression. An adapted form of ST for chronic depression has been described [17] and its efficacy has been demonstrated [18–20].

### Psychotherapy dosage

There are indications that psychotherapy dosage (operationalized as the number of sessions, treatment intensity or treatment duration) is a relevant factor for outcome in both personality disorders and depression. A meta-analysis in Cognitive Behavioral Analysis System of Psychotherapy (CBASP), designed for chronic depression and originally studied for 16 sessions, reports better outcomes when patients received more sessions [21]. For depression, most EST’s consist of 12–20 treatment sessions, but in more complex patients a higher number of sessions is not unusual, varying from 32 sessions in 1 year for patients with depression receiving ST [18] to 60 sessions of psychodynamic treatment within 18 months for patients with chronic depression and at least two failed treatment attempts [22]. For personality disorders, it is widely believed that more sessions are required [23–27] and most evidence based psychotherapies have a duration of at least 1 year. Nevertheless, Kool and colleagues [28] showed that not only depressive symptoms but also personality traits improved after only 16 sessions of SPSP in cluster-C patients. In addition, Bamelis et al. [16] found 50 sessions of ST to be more effective than treatment as usual in treating personality disorders and depression.

The frequency of sessions also appears to moderate effectiveness. A meta-analysis on session frequency in depressed patients found an effect size of d = .45 in favor of having twice weekly sessions versus once a week [29]. The effect of session frequency still needs to be tested directly in a randomized trial and an RCT comparing two dosages of Inter Personal Therapy (IPT) and Cognitive Behavioral Therapy (CBT) in a broad sample of depressed patients is now ongoing [30]. In PD, naturalistic studies focusing on intensity of treatment yield conflicting results. While Kordy and colleagues did not find an advantage of more frequent therapy sessions [26], others reported higher session frequency or treatment intensity to be related to better outcome [25, 31–33].

Taken together, the dosage issue seems to be a relevant factor in depressed patients and might be especially important when personality pathology underlies the (recurrent or chronic) depression. But it is unclear which treatment duration or intensity is needed to achieve enough improvement to prevent relapse.

### Objective

The main objective of the present study is to gather insight in the effect of treatment dosage on therapy outcome in patients meeting criteria of both a depressive disorder and PD, according to the Diagnostic Statistical Manual IV (DSM-IV) or -when available- 5 (DSM-5). We will compare two dosages of psychotherapy; 25 versus 50 sessions within a fixed period of 9 to 12 months in all conditions. SPSP and ST were selected as feasible approaches for this patient group in view of the available evidence for addressing depressive symptoms in the context of personality pathology. The primary hypothesis is that the 50 sessions condition is more effective in treating depression, as shown by a stronger reduction of depression severity and higher remission rates of depression.

Secondary we hypothesize a stronger reduction of susceptibility to depression in the 50 sessions condition by improvement of personality functioning as expressed at DSM-5 level, interpersonal functioning, schemas, schema modes and psychodynamic personality functioning. When personality vulnerabilities can be diminished during treatment, a sustained therapy effect is conceivable leading to less recurrence of depression. Cost-effectiveness will be evaluated at 1 year follow-up.

Additionally, secondary objectives will be addressed in this study in order to find therapy-specific and non-specific mechanisms of change. ST and SPSP have a different perspective on personality pathology: in ST PD are reflected in dysfunctional schemas and schema modes whereas in SPSP dysfunctional patterns in inter- and intrapersonal functioning reflect the personality problems. Although we expect improvement on all these measures in both treatment conditions, this enables us to explore whether the improvement of schemas and schema modes is larger in ST and improvement in inter- and intrapersonal functioning is larger in SPSP. Secondly, ST and SPSP are theoretically characterized by different working mechanisms: the presumed working mechanism of ST is the activation of dominant schemas reflected in schema modes during the sessions. It is explored to what extent this activation during the course of therapy precedes change. In SPSP adequate psychoanalytic support is the presumed working mechanism [34]. By measuring the subscales of the Working Alliance Inventory (WAI-sf) we will explore the aspects of the therapeutic alliance (agreement on goals, collaboration on tasks and therapeutic bond) as a mediator. SPSP can be placed on a variable point on the expressive-supportive continuum and interventions are adjusted to the level of insight the patient is capable of. We will check the hypothesis that the level of insight is not related to outcome. Finally, working alliance is a well-established mediator for change across all therapies [35] especially early measured working alliance [36]. In particular in this group of difficult to treat patients we expect a strong working alliance to be a predictor of positive treatment outcome in all conditions.

To our knowledge this is the first randomized dosage-effect-study for patients with both depression and PD. In addition, despite its high prevalence and clear indications that patients with depression and PD are more difficult to treat, effect-studies on tailored treatment modalities for this specific patient group are scarce.

## Methods/design

### Design of the study

A mono-center pragmatic randomized controlled trial with a 2 × 2 factorial design will be conducted with four parallel groups: 1) SPSP-25; 25 sessions of SPSP (*n* = 50), 2) ST-25; 25 sessions of ST (n = 50), 3) SPSP-50; 50 sessions of SPSP (n=50), 4) ST-50; 50 sessions of ST (n = 50). There is a fixed therapy duration of 9–12 months resulting in the same therapy-duration in all conditions, but different numbers of sessions and different session frequencies: in the 25-conditions once a week sessions during the first 4-6 months (16 sessions) will be followed by fortnightly sessions during the last 4-6 months (9 sessions). The 50-condition starts with a frequency of two sessions a week (32 sessions) followed by weekly sessions (18 sessions). The study follows a 2 × 2 factorial design that allows the comparison of dosage (25 vs 50 session in 1 year) in the combined therapy groups (ST + SPSP) and vice versa. The Medical Ethics Committee of VU University Amsterdam approved the study protocol (registration-number NL55916.029.15). The study is registered at the Netherlands Trial Register, part of the Dutch Cochrane Center (NTR5941).

### Participants

The study is conducted at the NPI, specialist in PD on two locations in Amsterdam (North and East). General criteria for referral are either insufficient results of earlier psychiatric or psychologic treatment or recurrence of depressive symptoms, clinically attributed to PD.

We aim to include 200 patients who meet the following criteria: 1) a DSM-5 depressive disorder (major depressive disorder or persistent depressive disorder) *and* one or more DSM-5 personality disorders, and 2) age 18–65 years. Patients will be excluded in case they: 1) are non-Dutch speakers/readers, 2) have psychotic symptoms, a bipolar disorder or current extreme substance dependence, 3) are in need of immediate and intensive treatment or hospitalization, e.g. acute suicidality according to the intake clinician, 4) are pregnant or unavailable for an uninterrupted period of more than 4 weeks 5) use medication which highly influences mental functioning according to a psychiatrist. No additional psychotherapy can be provided when participating in the trial, as this would exceed the assigned psychotherapy dosage. However, a psychiatrist can be consulted and pharmacotherapy can be provided if indicated. The consultation of psychiatrists and the prescribed pharmacotherapy during the trial will be monitored on the basis of information from the electronic prescription system as presented in Table 1. The Mini International Neuropsychiatric Interview-plus (MINI-plus) section A (Depression) and B (Dysthymia) will be used to diagnose depressive disorders until a version of the MINI-plus is available for DSM-5. The SCID-screener (SCID-PQ / SCID-5-SPQ) followed by the Structured Clinical Interview for DSM-IV personality disorders (SCID-II) or when available DSM-5 (SCID-5-PD) will be used to determine personality disorders.

**Table 1:** Monitoring of the pharmacotherapy delivered during the trial

Medication type	Dosage	Start date	End date
Antidepressants	ᅟ		
Benzodiazepinen			
Antipsychotics			
Other medication			

### Sample size

We powered the study to detect a difference of d = .45 between groups for depressive symptoms (BDI-II) [29] given the meta-analysis of session frequency in depressed patients described above, which reported an effect size of 0.45 when treatment sessions were given twice rather than once a week [29].According to this effect size (and given our choices of α = 0.05, two-tailed, power (1-β) = 0.80) 78 patients are needed in both dosage-groups. When 25% dropout is taken into account at least 200 patients will be needed for inclusion. With this sample size differences between SPSP and ST can be detected (with ≥80% certainty) if they differ at least d = .45.

### Recruitment

**Fig. 1:**
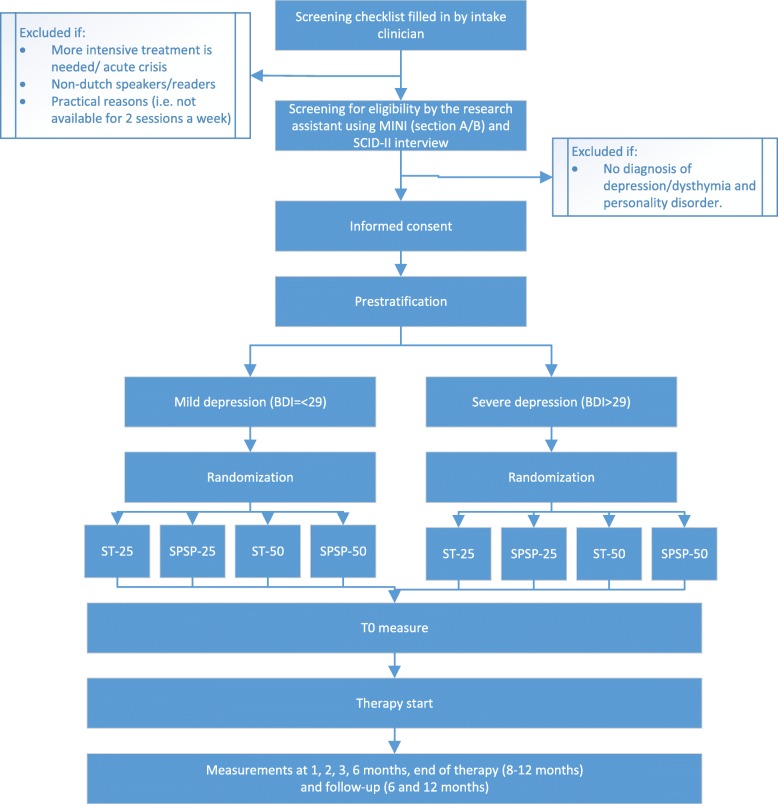
Trial flow diagram

Patients will be recruited from regular referrals to a mental health care center specialized in PD in Amsterdam (at two locations) in the Netherlands. Recruitment is planned for 2 years. During intake assessment, patients will be screened by the intake clinician on the in- and exclusion criteria. Patients who are eligible based on this screening will be approached by the research assistant. Patients who are excluded or not willing to participate will be referred to one of the regular treatment modalities by the intake clinician. In Fig. 1 an overview of the trial flow diagram is provided.

### Randomization and procedure

An independent research assistant will contact eligible patients within a week after intake and will invite the patient for research assessment. Additional information about the study is provided by email, giving the patient the time to consider participation. During the assessment meeting any doubts or questions about participating in the study can be discussed with the research assistant. The MINI-plus (section A and B) and the BDI-II will be assessed during research assessment and if no structured diagnostic interview has been completed during intake, the SCID-II / SCID-5-PD will be conducted. If the patient is eligible and willing to participate an informed consent is signed. Patients will then be randomized by one of two research department employees in one of four groups (with a 1:1:1:1 allocation) using a computer script performing block randomization (25 vs 50-sessions; ST vs SPSP). Randomization will be pre-stratified according to depression severity (BDI-II < =29 low, BDI-II= > 30 high). Treatment starts as soon as possible (approximately within 0 to 3 months) and patients are assigned to a therapist based on the allocated condition and the therapists availability. Patients will be assigned for the online baseline assessment in the week prior to the start of therapy. During the course of treatment patients will be requested to fill in short online assessments at 1, 2 and 3 months. At 6 months, end of treatment and follow-up extra questionnaires are added. The data collection process is monitored in detail and patients who don’t fill out the questionnaires will be reminded by the research assistant. In addition to the online measurements the SCID-II / SCID-5-PD and MINI-plus (section A and B) will be assessed at treatment termination (9–12 months) and follow-up (21–24 months) by a research assistant who is blind for condition. All data collected from interviews during research assessment at baseline, end of treatment and follow up will be entered and stored on site. Online submitted data forms are converted to the onsite database by the data analyst. All online submitted data will be checked by a research assistant on missing data or specific errors. Independent random data checks will be conducted on entry and encoding errors. All data is entered electronically. As part of the analysis, standard checks on double data entry and range checks on data values will be performed.

Therapists are requested to fill in online assessments at 1, 2, 3 and 6 months and at treatment termination concerning the therapeutic relation, the levels of discourse in SPSP and the dominant schema modes in ST. Participants may withdraw from the study for any reason at any time. Discontinuation of the treatment can also be indicated for serious clinical reasons as concluded in consultation meetings (i.e. immediate need for hospitalization or immediate need for an additional intervention leading to considerable exceedance of the assigned psychotherapy dosage). Early discontinuation of the treatment is not a reason for withdrawal from the study: these patients will be seen for an exit-interview and will be asked to continue participating in all remaining assessments. Participants have access to post-trial care if necessary for substantial clinical reasons during follow up period. An overview of all self-report measures and semi-structured interviews is presented in Table 2. All therapist and observer ratings per assessment are presented in Table 3.

**Table 2:** Overview of patient instruments per time point (months)

Instruments/months	I	A	0	1	2	3	6	END	FU 6 m	FU 12 m
Clinical outcomes										
Depression										
BDI-II		X	X	X	X	X	X	X	X	X
MINI plus, section A/B		X						X		X
Personality										
SIPP	X					X^a^	X	X	X	
SCID-screener	X							X		X
SCID-II / SCID-5-PD		X						X		X
YSQ-sf			X			X^b^	X	X		X
SMI			X			X^c^	X	X		X
DPI			X				X	X		X
Quality of life										
EQ-5D(5 L)			X			X	X	X		X
Happiness question			X	X	X	X	X	X	X	X
Cost-effectiveness										
Trimbos/iMTA Tic-P			X				X	X	X	X
Process/Predictors										
WAI-S				X	X	X	X	X		
Other measures										
Demografics	X									
Treatment history			X							
BSI	X		X			X	X	X		X
OQ-45	X					X	X	X		X

*I* Intake, *A* Assessment, *0* Start of therapy, *1/2/3/6* Months in therapy, *END* End of therapy (at 8–12 months), *FU 6 m* Follow-up at 6 month after end of therapy, *FU 12* Follow up at 12 months after end of therapy. ^a^SIPP subscale Relational Capacities, ^b^YSQ-sf subscales Emotional Deprivation and Failure, ^c^SMI subscales Healthy Adult, Happy Child and Detached Protector

**Table 3:** Overview of therapist and observer instruments per time point (months)

Instruments/months	I	A	0	1	2	3	6	END	FU 6 m	FU 12 m
Process/Predictors										
WAI-S				X	X	X	X	X		
Modes/Level of discourse				X	X	X	X	X		
Other measures										
End of treatment questionnaire								X		

*I* Intake, *A* Assessment, *0* Start of therapy, *1/2/3/6* Months in therapy, *END* End of therapy (at 8–12 months), *FU 6 m* Follow-up at 6 month after end of therapy, *FU 12* Follow up at 12 months after end of therapy

### Interventions

#### SPSP

SPSP is a supportive psychodynamic psychotherapy that uses a supportive stance and techniques to treat depression and to reduce depression vulnerability due to personality development. From a psychodynamic perspective SPSP emphasises the relational aetiology of and significance for the onset and recurrence of depressive symptoms. Current patterns of relationships are discussed and if appropriate related to interpersonal experiences that stem from the past and act as a mould for new relationships, both with others and oneself. The supportive stance and techniques contribute to the hypothesized working factor of SPSP: Adequate Psychoanalytic Support (APS). APS aims at fostering progression and countering regression by adequately gratifying unmet developmental needs in patients. SPSP has originally been tested for a broad group of depressed patients [37] in a dosage of 16 sessions. For patients with both depression and PD the therapy has now been intensified to either 25 or 50 sessions in 1 year.

**Fig. 2:**
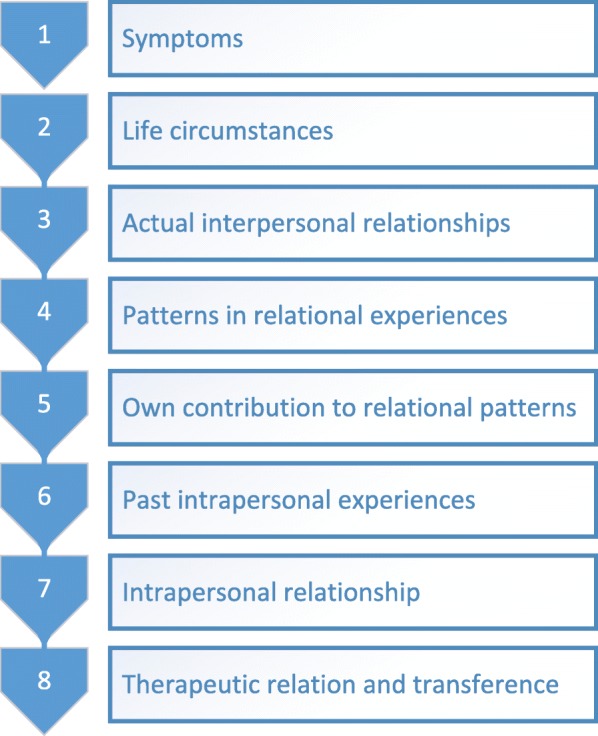
Levels of discourse in SPSP

In SPSP the therapist uses as a reference eight levels of discourse (i.e. levels of insight attainable by the patient) that serve to structure and foster the therapeutic process. As seen in Fig. 2 level one and two focus on the depressive symptoms and the influence of life circumstances on these symptoms. Interventions at these levels are mainly supportive, directed at behavioural activation, encouraging adaptive coping mechanisms, reducing feelings of guilt or giving praise or advice. At the third level the focus shifts to current relationships. At the fourth level patterns in these relations are discussed that may contribute to the onset or persistence of depressive feelings. Then, at the fifth level, the focus proceeds to the patient’s own contribution to the ongoing existence of these patterns. The sixth level focuses on an etiologic explanation to this contribution and how past relational experiences influence the patient’s current life. At the seventh level, the relationship the patient maintains with himself (for instance self-esteem regulation) is discussed as a consequence of identification with past internal-interpersonal relational experiences. Level eight concerns the relationship with the therapist and transference manifestation. At the higher levels the therapy is focusing more on personality functioning and the therapist uses more interventions to facilitate insight. The discourse levels are an attempt to structure the steps in the therapeutic process and to find the most helpful focus for the patient. The goal of SPSP is not to reach the highest level of insight, but to meet the patient on the level of insight he is capable of and which helps diminish the depression and improve interpersonal and intrapersonal functioning. SPSP can be placed on a variable point on the expressive- supportive continuum as the therapist is allowed to adopt a more supportive or more interpretative stance. In the second half of the therapy the frequency of the therapy sessions decreases, matching the developed growth, acquired insight and internalized adequate psychoanalytic support and stimulating the patient to integrate these in daily live (Fig. 2).

#### Schema therapy

Schema therapy is an integrative psychotherapy combining experiential, cognitive-behavioral, psychodynamic and interpersonal techniques [15]. Schemas can develop after basic emotional needs have not been met in early life. A schema mode is an emotional state in which one or more schemas are active and often has its particular coping strategy, once developed to protect oneself, but eventually causing more harm than good. The goal in ST is to develop the healthy adult mode by reducing harmful coping modes, battling the punitive (self-criticizing) and demanding modes and teaching patients how to meet and be met in their emotional needs. Following the development of ST, the focus in ST in the current study is on mode work [38]. The ST protocol for chronic depression [17] is used and adapted to fit the current study in terms of number of sessions and session frequency.

The therapy is divided into three phases. In the assessment and exploration phase the patient learns to understand the schema modes both cognitively and emotionally, their etiology and their interaction with depression. Techniques specifically used in this phase are psycho-education and diagnostic imaginations. Although a shorter period is often sufficient, this phase will take a maximum of 7 weeks (accounting for 7 weekly sessions in the 25 sessions condition and 14 twice weekly sessions in the 50 sessions condition).

In the second phase several techniques can be used to achieve change on cognitive, emotional and behavioral levels using cognitive techniques, experiential techniques such as chair work and imagery rescripting, behavioral techniques and techniques focusing on the therapeutic relationship such as empathic confrontation. The therapist has the role of a good parent, thereby providing a safer, warmer and more predictable environment than most patients have experienced early in life. As the healthy adult mode and the patients autonomy is expected to develop, session frequency is lowered at the second half of this therapy phase. This phase will take approximately 17 weeks (accounting for 9 weekly sessions, followed by 4 sessions every 2 weeks in the 25 sessions condition; 18 sessions twice weekly, followed by 8 weekly sessions in the 50 sessions condition).

**Fig. 3:**
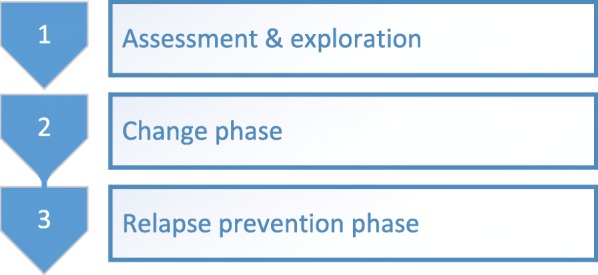
Phases in ST for depression

In the final phase of therapy behavioral change is aimed at and a relapse prevention plan will be developed in order to help the patient to get aware of risk factors for relapse and to address healthy actions when faced with these risk factors. Interventions are mainly focused on strengthening the healthy adult mode, autonomy and preparing for life after therapy. This last phase in therapy takes 10 weeks (accounting for 5 sessions every 2 weeks in the 25 sessions condition; 10 weekly sessions in the 50 sessions condition). An overview of the phase of the therapy and the corresponding session frequency is given in Fig. 3.

#### Therapists

All therapists have an academic degree in Psychology or Psychiatry and are health care psychologists, psychotherapists, clinical psychologists or psychiatrists. All participating schema therapists are registered schema therapists in the Netherlands or schema therapists in training. When not yet registered the therapist has at least 6 months experience in ST and receives additional supervision. A 1 day training in ST for depression was given to all therapists in the ST condition by a registered supervisor in ST. The SPSP therapists are registered as SPSP therapists or in training. When not yet registered the therapist has at least 6 months experience in SPSP and receives additional supervision. All registered therapists in de ST or SPSP condition can consult each other in biweekly consultation meetings. All sessions are audio-taped for both supervision- and adherence-purposes.

#### Instruments

At baseline screening a research-assistant (MSc student holding a Bachelors-degree in clinical psychology or junior researcher, holding a MSc degree in clinical psychology; trained in SCID-II / SCID-5-PD and MINI-plus assessments) will conduct semi-structured interviews to verify whether the patient meets the inclusion criteria by diagnosing a depressive disorder and PD. At treatment termination and follow-up these interviews are repeated by a research-assistant blind to condition. In order to detect treatment effect the SCID-II / SCID-5-PD interviews at treatment termination and follow-up will cover the previous 6 months. A check on interrater reliability will be conducted for both SCID-II / SCID-5-PD and MINI at baseline and follow-up.

#### Clinical outcomes measures

##### Primary outcome: depression

***Beck Depression Inventory (BDI-II-NL)*** [39]. The BDI-II is a self-report instrument of 21 items assessing depressive symptoms during the last 2 weeks. The BDI is a strong screening instrument for the severity of depressive symptoms [40, 41] and is widely used for clinical and research purposes. It has shown good psychometric qualities for both the original [42] and the Dutch version [43].

***Mini-International Neuropsychiatric Interview (MINI-plus) section A (depression) and B (dysthymia)*** [44, 45]. The MINI-plus was designed as a structured interview for the major Axis I psychiatric disorders in DSM-IV and ICD-10. Until a DSM-5 version of the MINI-plus is available, section A and B of the MINI-plus will be assessed in order to diagnose DSM-5 depressive disorders at screening, termination and follow-up. The MINI-plus has acceptably high validity and reliability scores [46].

##### Secondary outcome: personality parameters

***Structured Clinical Interview for DSM-IV personality disorders (SCID-II)*** [47, 48]. The SCID-II is a semi-structured interview used to diagnose DSM-IV personality disorders. Both the original and the Dutch version have adequate to good interrater reliability and test-retest reliability [49, 50]. Following the transitioning to the DSM-5, the SCID-II interview will be replaced by the SCID-5-PD interview [51] to diagnose DSM-5 personality disorders.

Assessments using the SCID-II / SCID-5-PD will be guided by disorders previously affirmed by the patient on the SCID-PQ / SCID-5-SPQ (cut-off-1), a self-report questionnaire screening for personality disorders [52] that will be completed before the interview. Disorders not affirmed on the SCID-PQ / SCID-5-SPQ will be assumed to be true negatives, however if the assessment clinician has reason to assume false negatives further items will be assessed. This method is in accordance with instructions for using the SCID-II / SCID-5-PD and enables the assessment of personality disorder symptoms to be based upon self-report combined with a structured clinical interview.

***Severity Indices of Personality Problems (SIPP-118)*** [53]. The SIPP-118 is a dimensional self-report questionnaire measuring the severity of personality pathology by assessing the core components of adaptive and maladaptive personality functioning. Overall, the psychometric features of the SIPP are good, with evidence for good reliability (α coefficients ranging from .62 to .89, with a mean estimated score of .78), convergent validity and invariance of the factor structure [53].

***Young Schema Questionnaire- short form (YSQ-sf)*** [54]. The YSQ-sf is a self-report instrument which is used to measure the presence or absence of 16 core maladaptive schemas. The YSQ has adequate temporal as well as rank-order stability. An analysis of its discriminant power in clinical versus non-clinical samples revealed that it is highly sensitive in predicting the presence or absence of psychopathology [55]. Internal consistency is high for the overall scale (Cronbach’s α ranges from .94 to .96) and satisfactory to high for its subscales (Cronbach’s α ranges from .72 to .94) [56]. Good to excellent psychometric properties were found for the Dutch version of the YSQ-short form, with high internal consistency for each subscale and a high level of concurrent and discriminative validity as described in an unpublished manuscript by Klynstra and Zwart.

***Schema Mode Inventory (SMI)*** [57]. The SMI is a self-report instrument that measures the extent to which 16 dysfunctional as well as functional schema modes are present [58]. Its subscales have satisfactory to high internal consistency (Cronbach’s α ranges from .79 to .96) and it is considered to be a useful instrument for assessing schema modes [59].

***Developmental Profile Inventory (DPI)*** [60]. The Developmental Profile Inventory (DPI) is a self-report instrument developed to assess psychodynamic personality functioning, based on the frame of reference of the so-called Development Profile (DP) [61]. The DPI was administered to patients in (day-) clinical psychotherapy (*N* = 179) and in a general population sample (*N* = 228). Internal consistencies of subsequent subscales are fair to good (.71 to .91 in healthy controls, .67 to .88 in the patient sample). Mean corrected item-total correlations were good (.30 to .50). Test-retest reliability was good to excellent (median ICC levels of .86 in healthy controls and .81 in the patient sample). The DPI also discriminated between patients and healthy controls in a meaningful way. Correlational analysis supported the distinction of two maladaptive clusters and a healthy adaptive cluster [60].

***Other secondary outcome measures***

Other secondary outcome measures are:

- Mental health and general psychiatric symptoms as measured with the ***Brief Symptom Inventory (BSI)*** [62] and the ***Outcome Questionnaire-45 (OQ-45)*** [63].
- General happiness as measured with the ***happiness item*** [64].
- Health care costs and lost productivity costs as measured with the ***Trimbos/iMTA questionnaire and Costs associated with Psychiatric illness (TiC-P)*** [65].
- Quality of life as measured with the ***EuroQol 5D (EQ-5D-5 L)*** [66].

#### Moderators and mechanisms of change

##### General patient characteristics

General patient characteristics will be collected at baseline, such as treatment history, DSM-IV/5 diagnoses, medication use, age, gender, marital status and educational level.

##### Treatment condition

The treatment condition, being either SPSP or ST will be reported in order to measure whether the effect of frequency is moderated by the type of therapy.

##### Level of discourse in SPSP

The dominant level of discourse will be reported by the SPSP-therapist at 1, 2, 3, 6 months and at end of treatment, ranging from level one focusing on physical and psychological symptoms to level eight focusing on the manifestation of the problems in the patient-therapist relation. This therapist-report scale will be developed for the study.

##### Dominant schema mode

The dominant schema mode active during the sessions will be reported by the ST-therapists at 1, 2, 3, 6 months and at end of treatment. Twelve dysfunctional and two functional modes can be selected. This therapist-report scale will be developed for the study.

##### Working alliance: working alliance inventory-short form [67, 68]

The WAI-sf intends to measure components of the therapeutic alliance by investigating the tasks, bonds and goals therapist and patient have. Behaviors and cognitions that form the therapeutic process, positive personal attachments between patient and therapist, and having set mutually endorsing and valuing goals are part of these components. The questionnaire consists of 12 items rated on a 5-point Likert scale and will be filled out by both patient and therapist. The instrument has shown adequate psychometric properties. It will be administered monthly in the first 3 months, at 6 months and at the end of therapy.

##### Therapist’s adherence

All sessions will be audio-taped and a planned selection of three sessions per patient (6–12%) will be scored by independent raters in order to measure therapists’ adherence to the treatment condition. For schema therapy an unpublished treatment integrity scale is used, developed for previous research [16]. Because no adherence scale for SPSP has been developed yet, this will be developed by SPSP-supervisors.

### Ethical and safety reporting

Serious adverse events (SAE; e.g. life-threatening events, resulting in permanent damage or death) will be documented throughout the study. In case of an SAE the principal investigator will be informed and will report the SAE to the local ethics committee. The ethics committee and the study team will then decide in accordance with the best interest of the patient if the study procedures are continued or terminated.

### Data handling and storage

At inclusion, a unique number will be allocated to each subject. The key of these numbers will only be available to the corresponding author and two data analysts who conduct randomization. All data will be stored encoded and in password protected files. Storage of data will be supervised by the principal investigator and complies with the Dutch Personal Data Protection Act (“WBP”).

### Data analyses

#### Clinical outcome: depression

Data-analysis will include an intention-to-treat analysis. Baseline characteristics and antidepressants used during treatment will be examined in the two dosage conditions (25 and 50 sessions) and potential confounding factors will be added as covariates in the analyses.

The primary outcome measure is depression severity (BDI-II). Relative effectiveness of the two dosage conditions (25- vs 50-sessions) will be analyzed using linear mixed models, with the underlying distributional model based on the distribution of the dependent variable and the residuals (e.g. normal, gamma, negative binomial). With this model missing values can be dealt with effectively. This analysis will be conducted according to a four-level structure (patient, therapist, location and time). The growth curve on depression severity over time can be shown with this model. The proportion of patients that achieve reliable and clinically significant improvement at termination and follow-up will be calculated on the outcome measure (BDI-II) for both dosage conditions, and secondary for both therapy-conditions (ST/SPSP). These calculations will be based on the reliable change index [69]. Comparison between the dosage conditions (and secondary between the therapy-conditions) will be analyzed with generalized estimating equations (GEE). In addition, remission rates will be measured and compared for the dosage conditions (and secondary for the therapy-conditions) at termination and follow-up (MINI-plus, section A and B) using GEE. In exploratory analyses, the interaction of treatment condition and dosage condition will be analyzed for all outcome measures.

#### Secondary outcome: personality parameters

Measuring change in personality traits in the course of both dosage and therapy conditions will be done using (generalized) linear mixed models. Also the percentage of patients reaching reliable and clinically significant improvement on personality outcomes will be analyzed for both dosage and therapy conditions, using the method of Jacobson and Truax [69]. Comparison between the dosage conditions (and secondary between the therapy-conditions) will be analyzed using GEE. Finally, remission rates of personality disorders will be measured and compared for both conditions (and secondary for the therapy-conditions) with GEE at termination and follow-up (SCID-II / SCID-5-PD).

#### Economic evaluation

The economic evaluation will be conducted alongside the randomized trial, taking into account the CHEERS statement [70] and the 2015 ISPOR good research practices task force report on cost-effectiveness analysis alongside clinical trials [71]. In the economic evaluation the difference in societal costs generated by patients in the two frequency conditions (25- vs 50-sessions) will be related to the difference in clinical effects. Both a cost-effectiveness and cost-utility analysis will be performed with a time horizon of 24 months. We will consider four types of costs: (1) the costs of offering the intervention (ST or SPSP, 25 or 50 sessions), (2) costs stemming from general health care uptake besides ST / SPSP, including the costs of medication, (3) patients’ out-of-pocket expenses (e.g. traveling costs, leisure time spent on receiving care), (4) costs stemming from productivity losses due to absenteeism or reduced efficiency while at work (presenteeism). The first two types of costs will be based on the full economic costs of offering the interventions. Here, we will use the standard cost prices reported in the Dutch guideline for economic evaluation [72]. Productivity losses will be based on the gender- and age-specific labour costs, and will be estimated using the friction cost approach. Data on resource use (health care uptake) and productivity losses will be collected using the TiC-P. Medication use will be valued using prices of the Royal Dutch Society for Pharmacy. Societal costs will be related to the following effect measures in the economic evaluation: 1. Decrease in depressive symptoms over 24 months as measured with the BDI-II (cost-effectiveness analysis), 2. Quality-adjusted life-years (QALY’s) measured over 24 months, based on the Dutch tariff for the EuroQol (EQ-5D) (cost-utility analysis).

The analyses will be done according to the intention-to-treat principle. Missing cost and effect data will be imputed using multiple imputation. Incremental cost-effectiveness-ratios (ICERs) will be calculated by dividing the difference in the mean total costs between the treatment groups by the difference in mean effects between the treatment groups. Uncertainty around the ICER will be calculated using a non-parametric bootstrap approach. For each of these bootstrapped samples, the incremental costs, incremental effects, and the ICER will be calculated. The resulting ICERs will be used for further calculations and will be plotted on a cost-effectiveness plane. In addition, cost-effectiveness acceptability curves (CEACs) will be constructed to show the probability that the 50-sessions intervention is cost-effective compared to the 25-sessions intervention, as a function of willingness-to-pay per additional unit of effect (QALYs or BDI symptoms). One-way sensitivity analyses directed at uncertainty in the main cost drivers will be performed to gauge the robustness of our findings.

### Drop out analysis

Treatment dropout rates will be compared between conditions using survival analysis.

### Analysis of moderators and mechanisms of change

To identify mechanisms of change and the strength of the factors involved, both multilevel mediation models and structural equation models will be used. Mixed regression analysis will be used to analyze the moderating effect of type of therapy (SPSP/ST) on outcome.

## Discussion

In this article the design of a pragmatic randomized clinical trial comparing two psychotherapy dosages is presented in a sample of patients with both depression and PD. This study is the first to investigate the effect of therapy dosage in this specific co-morbid patient group. If this study would show 50 sessions to be more effective than 25 sessions in treating depression and preventing relapse, this implies better and more enduring treatment results, less patient suffering and a reduction of societal and health care costs. An important additional aim of this study is to test a potential differential effect on improvement of personality characteristics between dosages. If the 50 sessions condition would lead to a more healthy development of personality characteristics, this could have protective value against relapse of depression. In addition, this study will contribute to the scarce evidence available on integrated therapies such as SPSP and ST in treating patients suffering from both depression and PD.

A long standing and poorly resolved important clinical question in psychotherapy is ‘what works for whom and why’. By addressing several pretreatment predictors, moderators and process factors we hope this study contributes to this question. Also, differential predictive factors may be found in ST and SPSP, by measuring both specific and non-specific treatment factors. However, single studies are generally underpowered to develop reliable prediction models. Combining data of studies in Individual Participant Meta-analyses is now recognized as a gold standard approach in prediction modeling research [73]. By gathering all data reliable and fully transparent we think this study may contribute to create this kind of joint analyses of data. In future this will allow us to select better optimal treatments for individual patients and shed more light on working mechanisms in psychotherapy.

Generalization of results from RCT designs are criticized because of, among other things, their limited inclusion and the often rigid conductance of therapies. In addition, we expected problems with acceptance by this complex patient sample of measurements at each session, and therefore a possible increase in the risk of dropout and a reduction of the generalizability of the study as a whole, limiting its value for daily clinical practice. This RCT is conducted in an ecologically valid environment of complex secondary care patients. We tried to make inclusion criteria as broad as possible. We will check for a good therapy reliance but both protocols allow a certain flexibility to adjust it to individual needs of the patients. They are not conducted according to a session by session description, as both treatment protocols prescribe adaptation of the focus of treatment and techniques to the momentary state of the patient. We hope this may facilitate participation and moderate dropout during therapy, which normally is relatively high in this kind of complex patient samples. Nevertheless, studying dosages makes it inevitable to apply a fixed number of sessions and session frequency. In clinical practice dosage might be applied with more flexibility depending on the course of therapy. Therefore, we think it would be helpful to complement data derived from this RCT by observational studies to accomplish a well-balanced evidence for clinical practice.

A strength of this study is the fact that patients are randomized over both dosage and type of therapy. This provides the opportunity to compare differences between both dosages in the larger samples of the combined therapy groups and vice versa. In addition, the large amount and broad range of both self-report and observer-rated measures of depression, personality, psychodynamic measures, and moderators and mediators are a strength of the study. Also, the frequent assessments on multiple time-points during therapy could give us the opportunity to detect temporal relationships that may shed light on working mechanisms. Moreover, an additional strength of this study is that it includes researchers with allegiance to both ST and SPSP, reducing the potential effects of researcher allegiance on treatment outcome. Finally, the economic evaluation is a strength of this study as it can gather insight in the effect of dosage on several aspects of costs.

The trial has several limitations that should be considered in evaluating the results. The first limitation of this study is the absence of a treatment as usual (TAU)- or waiting list condition. Since both conditions were adapted forms of existing therapies and were tailored for this subgroup of comorbidity, they have not been tested before in this form. However, we are dealing with a complex group of patients, almost all of whom have been treated earlier with little success, usually with therapies such as PST, CBT or IPT that are widely available in the Netherlands, and/or in combination with antidepressants. This made it difficult to find a feasible and acceptable TAU condition. In addition, all the patients were referred for specialized treatment, which, from a patient’s perspective, makes a prolonged stay on a waiting list unacceptable. As the duration of all the treatments is 1 year, we also considered it inappropriate to include a waiting list condition for the eligible patients. Instead, we chose to offer two specialized treatments focusing on depression in relation to underlying personality pathology. In addition, the main research question is the effect of a higher dosage of treatment. The 25 condition may therefore in effect be considered as a control condition. The effectiveness of both therapies in broad groups of depressed patients has been demonstrated [18, 74], in the case of PDT normally in 15–20 sessions and in the case of SFT in 32 sessions. We therefore chose the 25 condition as the minimum dosage required to be effective.

Secondly, one might question whether the two treatments are really different in daily practice. In general, common factors in the treatments, and in particular the therapeutic relationship, have been shown to be the most relevant working mechanisms for psychotherapy (e.g. [75]) and both therapies involve a search for underlying patterns related to the depression. Nevertheless, in practice, we think the approaches are perceptibly different at the level of applied concepts and techniques. In SFT, this difference is seen in the schemas and modes with the distinctive feature, in SPSP being the levels of discourse used to gain insight. Interventions such as diagnostic imaginations and experiential techniques such as role play and imagery rescripting are applied in SFT, while SPSP is a more open and exploratory discussion with the patient conducted in order to acquire an insight into the background of depression from an interpersonal and intrapersonal perspective. We therefore expect a difference in approach to be perceptible for both therapists and patients. This is also checked in the adherence part of the study.

The scope of this study does not allow for a full check of the differences between SPSP and SFT in terms of outcome. However, potential differences related in particular to the aims of the interventions could be analyzed at an exploratory level using secondary personality outcome measures. Outcome measures were therefore not only related to DSM-5 categories of personality disorders (SCID-PD) but also to both psychodynamic (SIIP-118, DPI) and schema therapy (YSQ-sf, SMI) concepts.

Thirdly, we used wide eligibility criteria in an attempt to select a sample which is representative for the general group of patients with depression and PD. Nevertheless, all patients included in the trial were referred to a center for PD assuming that the less severe and less enduringly depressed patients with PD might be missed out in the trial as they are treated at centers specialized in depression. Also, depression severity is used as the main outcome measure, even though this comorbid patient group has more psychological complaints than only depressive symptoms.

Fourthly, categorical DSM diagnoses were used to measure the inclusion criteria for both depression and PD. Thus nearly legitimate patients were excluded which, in view of the dimensional distribution in the real world of both severity personality pathology and of depressive symptoms, limits the generalization of the study.

Also, the dosage conditions of 25 or 50 sessions were chosen arbitrary and although these dosages can be considered as adequate for treating depression, the dosage is considered to be relatively low for treating PD. If both 25 and 50 sessions would not be sufficient to treat depression in this co-morbid patient group, it could be falsely interpreted that, without clear differences between the groups, the less intensive and less expensive 25 sessions condition would be preferred. Instead, effect sizes must be taken into account and additional research might be necessary to investigate whether higher dosages will lead to better outcomes.

The current study cannot distinguish between session frequency and total amount of sessions because the therapy duration was set to 1 year in all conditions. Thus, in case of significant differences in favor of the 50 sessions, follow-up studies are needed to disentangle the contribution of the number of sessions from the frequency effects.

A last limitation of the study is the fact that it is not powered to detect a clinically relevant difference between SPSP and ST if this would be smaller than d = .45. Even if the study would be powered to be able to detect a difference smaller than d = 0.45, we would not expect a difference between SPSP and ST. However, the study sample is not large enough and therefore underpowered to demonstrate equivalence between both theoretical treatment orientations.

This study aims to contribute to the evidence on psychotherapy for patients with co-occurring depression and personality disorders. Furthermore, this is the first study to compare two psychotherapy-dosages in this comorbid patient group. This study can help to get insight in how frequent and how much psychotherapy is needed to treat these complex patients. And finally, we hope this study will help to understand some of the processes that account for therapeutic changes.

### Trial status

The trial is in the ongoing recruitment phase.

## Abbreviations

APS: Adequate psychoanalytic support
BDI-II: Beck’s Depression Inventory II
BSI: Brief Symptom Inventory
CBASP: Cognitive Behavioral Analysis System of Psychotherapy
CBT: Cognitive Behavioral Therapy
CEACs: Cost-effectiveness acceptability curves
DPI: Developmental Profile Inventory
DSM-5: Diagnostic Statistical Manual 5
DSM-IV: Diagnostic Statistical Manual IV
EST: Empirically supported treatments
GEE: Generalized estimating equations
ICER: Incremental cost-effectiveness-ratio
IPT: Inter Personal Therapy
MINI-plus: Mini International Neuropsychiatric Interview-plus
OQ-45: Outcome Questionnaire-45
PD: Personality disorder
PSYDOS: Psychotherapy dosage
QALY: Quality-adjusted life-years
RCT: Randomized controlled trial
SCID-5-PD: Structured Clinical Interview for DSM-5 personality disorders
SCID-II: Structured Clinical Interview for DSM-IV Axis II disorders
SIPP-118: Severity Indices of Personality Problems – 118
SMI: Schema Mode Inventory
SPSP: Short-term Psychodynamic Supportive Psychotherapy
ST: Schema Therapy
TAU: Treatment as usual
TIC-P: Trimbos/iMTA questionnaire and Costs associated with Psychiatric illness
WAI-sf: Working Alliance Inventory- short form
YSQ-sf: Young Schema Questionnaire – short form

## References

[1] Friborg O, Martinsen EW, Martinussen M, Kaiser S, Overgard KT, Rosenvinge JH (2014). Comorbidity of personality disorders in mood disorders: a meta-analytic review of 122 studies from 1988 to 2010. J Affect Disord.

[2] Skodol AE, Stout RL, McGlashan TH, Grilo CM, Gunderson JG, Shea MT (1999). Co-occurrence of mood and personality disorders: a report from the collaborative longitudinal personality disorders study (CLPS). Depress Anxiety.

[3] Renner F, Bamelis LL, Huibers MJ, Speckens A, Arntz A (2014). The impact of comorbid depression on recovery from personality disorders and improvements in psychosocial functioning: results from a randomized controlled trial. Behav Res Ther.

[4] Svartberg M, Stiles TC, Seltzer MH (2004). Randomized, controlled trial of the effectiveness of short-term dynamic psychotherapy and cognitive therapy for cluster C personality disorders. Am J Psychiatry.

[5] Howard KI, Kopta SM, Krause MS, Orlinsky DE (1986). The dose-effect relationship in psychotherapy. Am Psychol.

[6] Soeteman DI, Hakkaart-van Roijen L, Verheul R, Busschbach JJ (2008). The economic burden of personality disorders in mental health care. J Clin Psychiatry..

[7] Soeteman DI, Verheul R, Busschbach JJ (2008). The burden of disease in personality disorders: diagnosis-specific quality of life. J Personal Disord.

[8] Newton-Howes G, Tyrer P, Johnson T, Mulder R, Kool S, Dekker J (2014). Influence of personality on the outcome of treatment in depression: systematic review and meta-analysis. J Personal Disord.

[9] Hellerstein DJ, Skodol AE, Petkova E, Xie H, Markowitz JC, Yen S (2010). The impact of comorbid dysthymic disorder on outcome in personality disorders. Compr Psychiatry.

[10] Leichsenring F, Schauenburg H (2014). Empirically supported methods of short-term psychodynamic therapy in depression - towards an evidence-based unified protocol. J Affect Disord.

[11] Driessen E, Cuijpers P, de Maat SC, Abbass AA, de Jonghe F, Dekker JJ (2010). The efficacy of short-term psychodynamic psychotherapy for depression: a meta-analysis. Clin Psychol Rev.

[12] Kool S, Schoevers R, de Maat S, Van R, Molenaar P, Vink A (2005). Efficacy of pharmacotherapy in depressed patients with and without personality disorders: a systematic review and meta-analysis. J Affect Disord.

[13] Fonagy P (2015). The effectiveness of psychodynamic psychotherapies: an update. World Psychiatry.

[14] Connolly Gibbons MB, Gallop R, Thompson D, Luther D, Crits-Christoph K, Jacobs J (2016). Comparative effectiveness of cognitive therapy and dynamic psychotherapy for major depressive disorder in a community mental health setting: a randomized clinical noninferiority trial. JAMA Psychiatry.

[15] Young JE, Klosko JS & Weishaar ME. Schema therapy: a practitioner’s guide. New York: Guilford Press; 2003.

[16] Bamelis LL, Evers SM, Spinhoven P, Arntz A (2014). Results of a multicenter randomized controlled trial of the clinical effectiveness of schema therapy for personality disorders. Am J Psychiatry.

[17] Renner F, Arntz A, Leeuw I, Huibers M (2013). Treatment for chronic depression using schema therapy. Clin Psychol Sci Pract.

[18] Carter JD, McIntosh VV, Jordan J, Porter RJ, Frampton CM, Joyce PR (2013). Psychotherapy for depression: a randomized clinical trial comparing schema therapy and cognitive behavior therapy. J Affect Disord.

[19] Malogiannis IA, Arntz A, Spyropoulou A, Tsartsara E, Aggeli A, Karveli S (2014). Schema therapy for patients with chronic depression: a single case series study. J Behav Ther Exp Psychiatry.

[20] Renner F, Arntz A, Peeters FP, Lobbestael J, Huibers MJ (2016). Schema therapy for chronic depression: results of a multiple single case series. J Behav Ther Exp Psychiatry.

[21] Negt P, Brakemeier EL, Michalak J, Winter L, Bleich S, Kahl KG (2016). The treatment of chronic depression with cognitive behavioral analysis system of psychotherapy: a systematic review and meta-analysis of randomized-controlled clinical trials. Brain Behav.

[22] Fonagy P, Rost F, Carlyle JA, McPherson S, Thomas R, Pasco Fearon RM (2015). Pragmatic randomized controlled trial of long-term psychoanalytic psychotherapy for treatment-resistant depression: the Tavistock adult depression study (TADS). World Psychiatry.

[23] Leichsenring F, Rabung S (2011). Long-term psychodynamic psychotherapy in complex mental disorders: update of a meta-analysis. Br J Psychiatry.

[24] Perry JC, Banon E, Ianni F (1999). Effectiveness of psychotherapy for personality disorders. Am J Psychiatry.

[25] Freedman N, Hoffenberg JD, Vorus N, Frosch A (1999). The effectiveness of psychoanalytic psychotherapy: the role of treatment duration, frequency of sessions, and the therapeutic relationship. J Am Psychoanal Assoc.

[26] Kordy H, von M R, Senf W (1988). Time and its relevance for a successful psychotherapy. Psychother Psychosom.

[27] Lambert JJO, B.M. The efficacy and effectiveness of psychotherapy. In: L MJ, editor. Bergin and Gafield’s handbook of psychotherapy and behavior change. New York: Wiley; 2013.

[28] Kool S, Dekker J, Duijsens IJ, de J F, Puite B (2003). Changes in personality pathology after pharmacotherapy and combined therapy for depressed patients. J Personal Disord.

[29] Cuijpers P, Huibers M, Ebert DD, Koole SL, Andersson G (2013). How much psychotherapy is needed to treat depression? A metaregression analysis. J Affect Disord.

[30] Bruijniks SJ, Bosmans J, Peeters FP, Hollon SD, van O P, van den B M (2015). Frequency and change mechanisms of psychotherapy among depressed patients: study protocol for a multicenter randomized trial comparing twice-weekly versus once-weekly sessions of CBT and IPT. BMC Psychiatry.

[31] Reardon ML, Cukrowicz KC, Reeves MD, Joiner TE (2002). Duration and regularity of therapy attendance as predictors of treatment outcome in an adult outpatient population. Psychother Res.

[32] Bartak A, Spreeuwenberg MD, Andrea H, Holleman L, Rijnierse P, Rossum BV (2010). Effectiveness of different modalities of psychotherapeutic treatment for patients with cluster C personality disorders: results of a large prospective multicentre study. Psychother Psychosom.

[33] Soeteman DI, Verheul R, Meerman AM, Ziegler U, Rossum BV, Delimon J (2011). Cost-effectiveness of psychotherapy for cluster C personality disorders: a decision-analytic model in the Netherlands. J Clin Psychiatry.

[34] De Jonghe F, De Maat S, Hendriksen M, Nooteboom A, Dekker J, Van HL (2013). Short-term psychoanalytic supportive psychotherapy for depression. Psychoanal Inq.

[35] Martin DJ, Garske JP, Davis MK (2000). Relation of the therapeutic alliance with outcome and other variables: a meta-analytic review. J Consult Clin Psychol.

[36] Hendriksen M, Peen J, Van R, Barber JP, Dekker J (2014). Is the alliance always a predictor of change in psychotherapy for depression?. Psychother Res.

[37] Driessen E, Van HL, Don FJ, Peen J, Kool S, Westra D (2013). The efficacy of cognitive-behavioral therapy and psychodynamic therapy in the outpatient treatment of major depression: a randomized clinical trial. Am J Psychiatry.

[38] Arntz A, Jacob G (2012). Schema therapy in practice.

[39] Beck AT, Steer RA, Brown GK, van der Does AJW (2016). BDI-II-NL-R Handleiding.

[40] Beck AT, Steer RA, Brown G (1996). Manual for the Beck depression inventory-second edition (BDI-II).

[41] Whisman MA, Perez JE, Ramel W (2000). Factor structure of the Beck depression inventory-second edition (BDI-II) in a student sample. J Clin Psychol.

[42] Beck AT, Steer RA, Carbin MG (1988). Psychometric properties of the Beck depression inventory: twenty-five years of evaluation. Clin Psychol Rev.

[43] Luteijn F, Bouman TK (1988). De validiteit van Beck’s Depression Inventory. Nederlands Tijdschrift voor de Psychologie.

[44] Vliet IM, Leroy H, Megen HJGM (2000). MINI international neuropsychiatric interview (M.I.N.I.). Nederlandse versie 5.0.0 ed.

[45] Lecrubier Y, Sheehan DV, Weiller E, Amorim P, Bonora I, Harnett Sheehan K (1997). The MINI international neuropsychiatric interview (MINI). A short diagnostic structured interview: reliability and validity according to the CIDI. Eur Psychiatry.

[46] Sheehan DV, Lecrubier Y, Sheehan KH, Amorim P, Janavs J, Weiller E (1998). The Mini-International Neuropsychiatric Interview (M.I.N.I.): the development and validation of a structured diagnostic psychiatric interview for DSM-IV and ICD-10. J Clin Psychiatry.

[47] First MB, Spitzer RL, Gibbon M, Williams JBW (1997). Structured clinical interview for DSM-IV Axis II personality disorders, (SCID-II).

[48] Weertman A, Arntz A, Kerkhofs MLM. Gestructureerd Klinisch Interview voor DSM-IV As-II Persoonlijkheidsstoornissen (SCID-II): Lisse. Swets Test Publishers; 2000.

[49] Lobbestael J, Leurgans M, Arntz A (2011). Inter-rater reliability of the structured clinical interview for DSM-IV Axis I disorders (SCID I) and Axis II disorders (SCID II). Clin Psychol Psychother.

[50] Maffei C, Fossati A, Agostoni I, Barraco A, Bagnato M, Deborah D (1997). Interrater reliability and internal consistency of the structured clinical interview for DSM-IV axis II personality disorders (SCID-II), version 2.0. J Personal Disord.

[51] First MB, Williams JB, Benjamin LS, Spitzer RL (2016). Structured clinical interview for DSM-5 personality disorders: SCID-5-PD.

[52] First MB, Williams JB, Benjamin LS, & Spitzer RL. SCID-5-SPQ: Structured Clinical Interview for DSM-5 Screening Personality Questionnaire: Designed to be Used as a Screener for the Structured Clinical Interview for DSM-5 Personality Disorders (SCID-5-PD). Arlington: American Psychiatric Association Publishing; 2016.

[53] Verheul R, Andrea H, Berghout CC, Dolan C, Busschbach JJ, van der Kroft PJ (2008). Severity indices of personality problems (SIPP-118): development, factor structure, reliability, and validity. Psychol Assess.

[54] Rijkeboer MM (2008). Schema-Vragenlijst Verkorte Vorm.

[55] Rijkeboer MM, van den B H, van den B J (2005). Stability and discriminative power of the young Schema-questionnaire in a Dutch clinical versus non-clinical population. J Behav Ther Exp Psychiatry.

[56] Baranoff J, Oei TP, Cho SH, Kwon SM (2006). Factor structure and internal consistency of the young Schema questionnaire (short form) in Korean and Australian samples. J Affect Disord.

[57] Young JE, Arntz A, Atkinson T, Lobbestael J, Weishaar M, Vreeswijk M (2007). The Schema mode inventory (SMI).

[58] Lobbestael J, Van Vreeswijk MF, Arntz A (2008). An empirical test of schema mode conceptualizations in personality disorders. Behav Res Ther.

[59] Lobbestael J, van V M, Spinhoven P, Schouten E, Arntz A (2010). Reliability and validity of the short Schema mode inventory (SMI). Behav Cogn Psychother.

[60] Polak MG, Van Riel L, Ingenhoven TJ, & Van HL. The Developmental Profile Inventory: Constructing a clinically useful self-report for levels of psychodynamic personality functioning. Journal of Psychiatric Practice. 2018;24(4):239-252. 10.1097/PRA.0000000000000323 PMC6278880 30427807

[61] Polak M, Van HL, Overeem-Seldenrijk J, Heiser WJ, Abraham RE (2010). The developmental profile: validation of a theory-driven instrument for personality assessment. Psychother Res.

[62] Beurs E (2004). Brief symptom inventory. Handleiding.

[63] Jong dK, Nugter MA, L MJ, Burlingame GM (2009). Handleiding voor afname en scoring van de Outcome Questionaire OQ-45.2.

[64] Veenhoven R (2014). Happiness Questionnaire.

[65] Hakkaart-van Roijen L, van Straten A, Donker M, Tiemens B. Trimbos/iMTA questionnaire for costs with psychiatric illness (TIC-P): Rotterdam. Institute for Medical Technology Assessment; 2002.

[66] Brooks R, Rabin R, de Charro F (2003). The measurement and valuation of health status using EQ-5D: a European perspective.

[67] Horvath AO, Greenberg LS (1989). Development and validation of the working alliance inventory. J Couns Psychol.

[68] Busseri MA, Tyler JD (2003). Interchangeability of the working alliance inventory and working alliance inventory. Short Form Psychol Assess.

[69] Jacobson NS, Truax P (1991). Clinical significance: a statistical approach to defining meaningful change in psychotherapy research. J Consult Clin Psychol.

[70] Husereau D, Drummond M, Petrou S, Carswell C, Moher D, Greenberg D (2013). Consolidated health economic evaluation reporting standards (CHEERS) statement. BMJ.

[71] Ramsey SD, Willke RJ, Glick H, Reed SD, Augustovski F, Jonsson B (2015). Cost-effectiveness analysis alongside clinical trials II-an ISPOR good research practices task force report. Value Health.

[72] Hakkaart-van Roijen L, van der Linden N, Bouwmans C, Kanters T, Tan SS. Kostenhandleiding: methodologie van kostenonderzoek en referentieprijzen voor economische evaluaties in de gezondheidszorg. Rotterdam: Institute for Medical Technology Assessment, Erasmus Universiteit Rotterdam; 2015.

[73] Debray TP, Vergouwe Y, Koffijberg H, Nieboer D, Steyerberg EW, Moons KG (2015). A new framework to enhance the interpretation of external validation studies of clinical prediction models. J Clin Epidemiol.

[74] Driessen E, Hegelmaier LM, Abbass AA, Barber JP, Dekker JJ, Van HL (2015). The efficacy of short-term psychodynamic psychotherapy for depression: a meta-analysis update. Clin Psychol Rev.

[75] Norcross JC, Wampold BE (2011). Evidence-based therapy relationships: research conclusions and clinical practices. Psychotherapy (Chic).

