# Correction: Optimizing psychotherapy dosage for comorbid depression and personality disorders (PsyDos): a pragmatic randomized factorial trial using schema therapy and short-term psychodynamic psychotherapy

**DOI:** 10.1186/s12888-023-04920-8

**Published:** 2023-06-16

**Authors:** Marit Kool, Henricus L. Van, Anna Bartak, Saskia C. M. de Maat, Arnoud Arntz, Johanna W. van den Eshof, Jaap Peen, Matthijs Blankers, Judith E. Bosmans, Jack J. M. Dekker

**Affiliations:** 1 Arkin Mental Health Care, Domselaerstraat 128, 1093 MB Amsterdam, the Netherlands; 2 Department of Clinical Psychology, University of Amsterdam, Amsterdam, the Netherlands; 3 Department of Research, Arkin Mental Health Care, Amsterdam, the Netherlands; 4 Amsterdam UMC, Location AMC, Department of Psychiatry, University of Amsterdam, Amsterdam, the Netherlands; 5 Trimbos Institute – Netherlands Institute of Mental Health and Addiction, Utrecht, the Netherlands; 6 Department of Health Sciences, Faculty of Earth & Life Sciences, Free University Amsterdam, Amsterdam Public Health Research Institute, Amsterdam, The Netherlands; 7 Department of Clinical Psychology, VU University of Amsterdam, Amsterdam, the Netherlands

**Correction: BMC Psychiatry 18, 252 (2018)**

**https://doi.org/10.1186/s12888-018-1829-1**

Following publication of the original article [1], the author identified errors in block randomization and sample size. The correct sentence of block randomization and sample size is given below.

Block randomization

The sentence currently reads:

Patients will then be randomized by one of two research department employees in one of four groups (with a 1:1:1:1 allocation) using a computer script performing block randomization (25 vs 50-sessions; ST vs SPSP).

The sentence should read:

Patients will then be randomized by one of two research department employees in one of four groups (with a 1:1:1:1 allocation) with random allocation sequences that were generated using the SPSS random number generator (SPSS, Chicago).

Sample size

The sentence currently reads:

According to this effect size (and given our choices of α = 0.05, two-tailed, power (1-β) = 0.80) *78* patients are needed in both dosage-groups. When 25% dropout is taken into account at least *200* patients will be needed for inclusion.

The sentence should read:

According to this effect size (and given our choices of α = 0.05, two-tailed, power (1-β) = 0.80) 79 patients are needed in both dosage-groups. When 25% dropout is taken into account at least 211 patients will be needed for inclusion.
